# Analysis of High-altitude Syndrome and the Underlying Gene Polymorphisms Associated with Acute Mountain Sickness after a Rapid Ascent to High-altitude

**DOI:** 10.1038/srep38323

**Published:** 2016-12-16

**Authors:** Jie Yu, Ying Zeng, Guozhu Chen, Shizhu Bian, Youzhu Qiu, Xi Liu, Baida Xu, Pan Song, Jihang Zhang, Jun Qin, Lan Huang

**Affiliations:** 1 Institute of Cardiovascular Diseases of PLA, Xinqiao Hospital, Third Military Medical University, Chongqing, China

## Abstract

To investigated the objective indicators and potential genotypes for acute mountain sickness (AMS). 176 male subjects were evaluated for symptoms scores and physiological parameters at 3700 m. EPAS1 gene polymorphisms were explored and verified effects of potential genotypes on pulmonary function by inhaled budesonide. The incidence of AMS was 53.98% (95/176). The individuals who suffered from headache with anxiety and greater changes in heart rate (HR), the forced vital capacity (FVC), and mean flow velocity of basilar artery (Vm-BA), all of which were likely to develop AMS. The rs4953348 polymorphism of EPAS1 gene had a significant correlation with the SaO2 level and AMS, and a significant difference in the AG and GG genotype distribution between the AMS and non-AMS groups. The spirometric parameters were significantly lower, but HR (P = 0.036) and Vm-BA (P = 0.042) significantly higher in the AMS subjects with the G allele than those with the A allele. In summary, changes in HR (≥82 beats/min), FVC (≤4.2 Lt) and Vm-BA (≥43 cm/s) levels may serve as predictors for diagnosing AMS accompanied by high-altitude syndrome. The A allele of rs4953348 is a protective factor for AMS through HR and Vm-BA compensation, while the G allele may contribute to hypoxic pulmonary hypertension in AMS.

In response to a rapid ascent to altitudes above 2500 m, the arterial oxyhemoglobin saturation (SaO2) decreases quickly due to hypobaric hypoxia and results in a series of discomforts, with acute mountain sickness (AMS) being the most common syndrome that occurs in unacclimated individuals^1^. The incidence of AMS is as high as 50% when individuals are exposed to high altitude^2^. Unlike other sicknesses that require complex tests, AMS is diagnosed based on the subjective reporting of symptoms. The Lake Louise consensus scoring system (LLS), the most commonly applied criteria for diagnosing AMS, depends on the subject’s own description of symptom severity, including headache, nausea or anorexia, weakness or dizziness, insomnia, and shortness of breath^3^,^4^,^5^, but these symptoms may not belong to a single syndrome. Headache is the primary symptom of AMS and associated with one or more other symptoms in the LLS diagnostic criteria. The changes in oxygenation, cerebral blood flow, and intracranial hypertension induced by hypoxic vasodilatation, can disrupt the blood-brain barrier and lead to headache in subjects with AMS^5^,^6^,^7^,^8^,^9^. Subjects susceptible to AMS may encounter impaired pulmonary function with a low hypoxic ventilator response (HVR) and an exaggerated hypoxic pulmonary vascular response (HPVR)^10^,^11^,^12^,^13^,^14^.

In addition, exposure to high altitude may also lead to psychological changes. Anxiety, the most prevalent mood state associated with AMS^15^,^16^,^17^,^18^, can cause physical discomforts, such as headache and insomnia^15^,^19^. Individuals with headaches are more likely to suffer from insomnia, and transient recurrent situational insomnia is related to headache^20^. At high altitude, sleep alterations can aggravate anxiety and are strongly associated with AMS^15^.

Because diagnosis of AMS based on the subjective reporting of symptoms, it is necessity for more objective criteria. Currently, there is a lack of detailed descriptions of the physiological and psychological symptoms upon acute exposure to high altitude. Furthermore, how these symptoms interact together and the relationship among the symptoms, commonly implicated physiological parameters and AMS are also unknown. More importantly, few researches emphasize the molecular mechanisms underlying adaptation to high-altitude hypoxia was and some investigation closely related it to the hypoxia-inducible factor (HIF) family of transcription factors, especially HIF2α (or EPAS1)^21^,^22^. However, few studies have been conducted on genetic susceptibility to AMS in individuals from low altitudes.

In this study, we conducted a cohort study to evaluate the effectiveness of the symptoms (including headache, insomnia, and anxiety), and the alterations in the levels of heart rate (HR), left ventricle (LV) Tei index, spirometric parameters, blood flow of the basilar artery (BA) and the vertebral artery (VA), mean arterial blood pressure (MABP), and angiotensin-converting enzyme (ACE) levels in AMS development. Furthermore, we explored the relationships between high-altitude syndrome and AMS occurrence, coupled with EPSA1 (rs2044456 and rs4953348) polymorphisms and AMS, and verified the effect of the potential genotypes on the pulmonary function by inhaled budesonide in the AMS subjects^23^. In summary, the purpose of this study were as follows: (1) to determine high-altitude syndrome and the objective indicators for AMS diagnosis, (2) to evaluate the effect of ascent to high altitude on HR, the forced vital capacity (FVC), and mean flow velocity of the basilar artery (Vm-BA) levels and determine whether the levels contribute to AMS prediction, and (3) to correlate the potential genotypes with AMS development.

## Results

### Comparison of physiological parameters between subjects at 500 m and 3700 m

**Figure 1:**
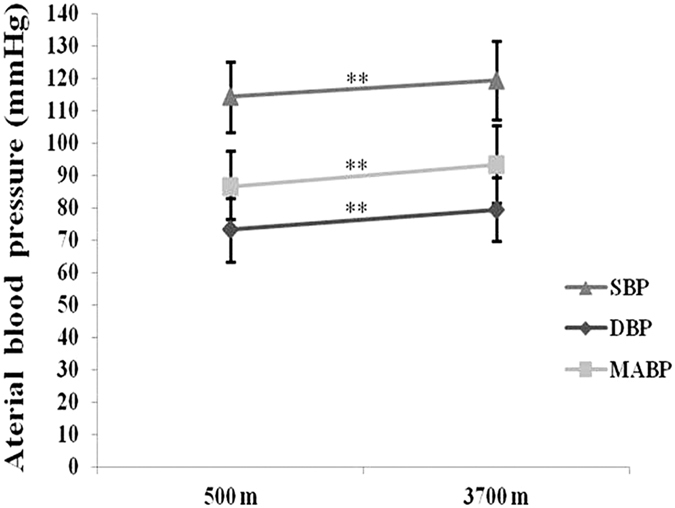
Changes in arterial blood pressure from 500 m to 3700 m. SBP, systolic blood pressure; DBP, diastolic blood pressure; MABP, mean arterial blood pressure. **P < 0.01.

All comparison data in subjects between levels of 500 m and 3700 m were present in Supplementary Table 1. With regard to vital signs, the SaO_2_ level was significantly lower at 3700 m levels (adjusted P < 0.01, Table 1), while the HRs and BP, including systolic blood pressure (SBP), diastolic blood pressure (DBP) and mean arterial blood pressure (MABP), increased significantly at 3700 m compared with 500 m (P < 0.01, Table 1 and Fig. 1). At 3700 m, the participants had significantly higher values of Hb and MCHC and significantly lower values of HCT (Table 1). With respect to left heart function, only significantly higher levels of EF (adjusted P < 0.05, Table 1) observed in subjects from 500 to 3700 m, although stroke volume (SV), stroke volume index (SVI), cardiac output (CO), cardiac index (CI) and the Tei index were measured higher in original data of 3700 m. Additionally, we evaluated parameters of right heart function. The mean pulmonary artery pressure (mPAP) (Supplementary Figure 1A), the tricuspid E/A ratio (Supplementary Figure 1B) and right atrium (RA) diameter (Supplementary Figure 1C) were higher at 3700 m, but disappeared after Bonferroni adjust (Supplementary Table 1). Compared with the levels at 500 m, the respective serum concentrations of epinephrine, norepinephrine, Ang II and Ang-(1–7) were increased significantly after acute exposure to 3700 m (P < 0.01, Table 1). No symptom score were found differences after adjust. With regard to brain function, the peak systolic flow velocity (Vs), diastolic velocity (Vd), and mean flow velocity (Vm) of the middle cerebral artery (MCA), basilar artery (BA) and vertebral artery (VA) increased and the pulsatility index (PI) and resistance index (RI) of the MCA, BA and VA decreased at 3700 m compared with the levels at 500 m, but such effects disappeared after Bonferroni adjust (Supplementary Table 2).

### Comparison of symptoms and clinical characteristics between the AMS and non-AMS groups at 3700 m

**Figure 2:**
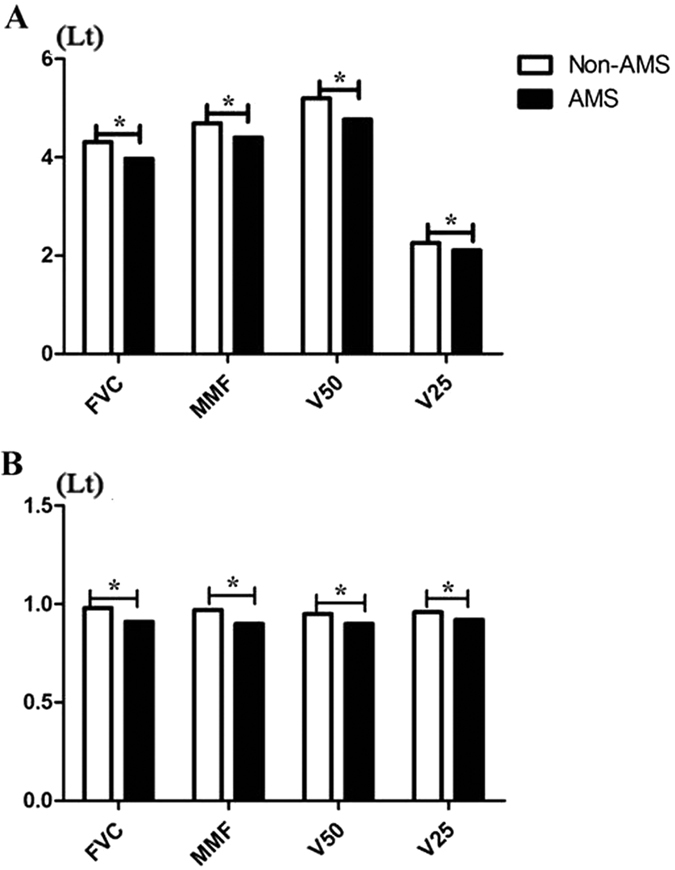
(**A**) Comparison between spirometric parameter and AMS at 3700 m; (**B**) The comparison between the range of changes in spirometric parameter and AMS after acute exposure to 3700 m. *P < 0.05.

Among the 176 recruits, 95 were diagnosed with AMS after acute exposure to 3700 m. No significant differences in demographic data, including age, height, BMI, smoking and alcohol consumption, were identified between two groups (Table 2). The incidence of the total symptoms, including the five symptoms of LLS, insomnia and anxiety, was significantly higher in the AMS group than in the non-AMS group (adjusted P < 0.01, Supplementary Table 3). With regard to vital signs, the AMS group had a trend of lower SaO2 level, and higher HRs and MABP than the non-AMS group, although such difference disappear after adjust (Supplementary Table 3). In terms of the cardiovascular and cerebrovascular systems, the Tei index of the heart had a trend of increase, and the LVET decrease in the AMS group compared with the non-AMS group (Supplementary Table 3). Furthermore, we measured isovolumetric contraction time (ICT), ejection time (ET), and isovolumetric relaxation time (IRT) using a pulsed Doppler diagram to calculate the Tei index (Supplementary Figure 2A). In the AMS group, a prolonged IRT and a shortened ET resulted in an increased Tei index (Supplementary Figure 2B), while the IRT and ET remained unchanged in the non-AMS group, causing no Tei index alteration (Supplementary Figure 2C). In contrast, the AMS group had a trend of higher Vs, Vd, and Vm in the BA; Vd, ΔVs, ΔVd and ΔVm in the VA; and percentage of AIVA than the non-AMS group. (Supplementary Table 3). The index at rest in the AMS and non-AMS groups is presented in Fig. 2A. The AMS group showed significantly lower forced vital capacity (FVC), maximal midexpiratory flow (MMF), and vital capacity (25–50)% (V25-50) compared with the non-AMS group (P < 0.05) (Fig. 2A).

### Indicators and predictors for an AMS diagnosis

**Figure 3:**
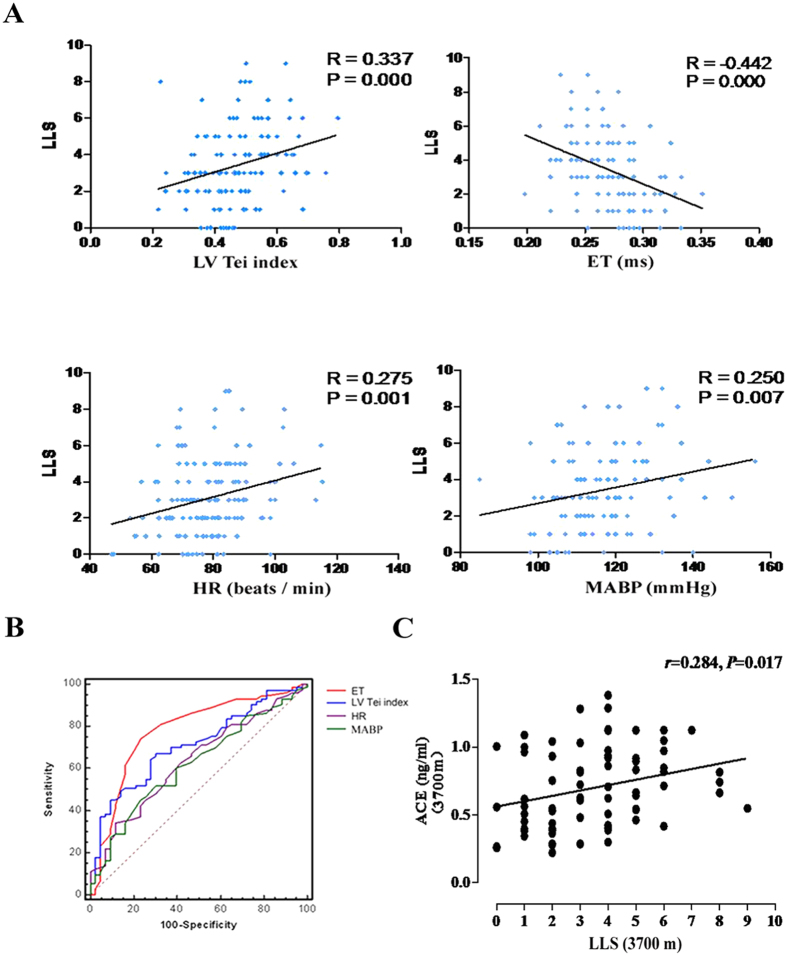
Indicators of acute mountain sickness (AMS). (**A**) Correlations of the left ventricular (LV) Tei index, ejection time (ET), heart rate (HR) and mean pulmonary arterial pressure (MABP) with the Lake Louise score (LLS) in all subjects under acute high altitude/hypoxic conditions. (**B**) Receiver operating characteristic (ROC) curve analysis of the ET (red line), LV Tei index (blue line), HR (purple line) and MABP (green line) was performed to test the diagnostic value of each parameter for AMS diagnosis. (**C**) Positive correlation between individual changes in serum ACE levels (ng/ml) and LLS after acute exposure to the high altitude (3700 m).

The indicators that can be used to diagnose AMS are based on the correlations between the physiological parameters and LLS, as assessed by linear regression analysis and spearman rank correlation analysis. First, the ranges of changes in spirometric parameter, including FVC, MMF, and V25-50, were associated with AMS (P < 0.05) (Fig. 2B). Second, an increased LV Tei index (R = 0.337, P = 0.000), HR (R = 0.275, P = 0.001), and MABP (R = 0.250, P = 0.007) and decreased ET (R = −0.442, P = 0.000) showed significant correlations with LLS (Fig. 3A). Moreover, receiver operating characteristic (ROC) curve analysis of the ET, LV Tei index, HR and MABP was performed to test the diagnostic value of each parameter for AMS diagnosis (Fig. 3B). Third, increased serum concentrations of ACE positively and significantly correlated with LLS (R = 0.284, P = 0.017) (Fig. 3C). The PI and RI of the MCA, BA and VA were significantly negatively correlated with LLS, while the Vm, Vs, and Vd of BA and the Vm, Vd, ΔVm, ΔVd, and AI of VA were positively correlated (Supplementary Table 4). Furthermore, the above mentioned factors and the symptoms including headache (OR: 3.15; 95%CI: 1.65–5.30; P < 0.001), anxiety (OR: 1.96; 95%CI: 1.32–3.61) and insomnia (OR: 0.78; 95%CI: 1.45–4.09; P < 0.001) were the independent factors for AMS and they were all strongly associated with AMS (Table 3). A logistic regression analysis revealed that HR, FVC, and Vm-BA can be considered as predictors for AMS. The possibility of AMS occurrence significantly increased when the subjects with HR ≥ 82 beats/min, FVC ≤ 4.2 Lt, and Vm-BA ≥ 43 cm/s simultaneously (P = 0.007) (Table 4).

### Genetic analysis and the correlation with clinical data

**Figure 4:**
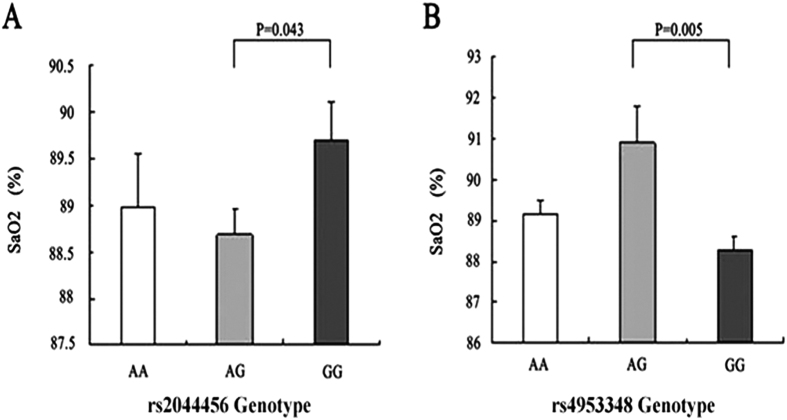
The correlation between AMS and the genotypes of rs2044456 (**A**) and rs4953348 (**B**). Significant differences in the SaO2 level were detected in the EPAS1 (rs2044456 and rs4953348) SNP genotypes (P = 0.043 and P = 0.005, respectively).

DNA sequencing was used to detect the AA, AG, and GG genotypes in the rs2044456 and rs4953348 polymorphism loci between the two groups (Supplementary Figure 3). As shown in Table 5, no significant differences in the genotype and allele frequencies of rs2044456 were identified between the two groups, while the AG and GG genotype distributions of rs4953348 showed significant differences in both groups (OR = 0.453, 95%CI 0.226–0.908, P = 0.026, adjusted P = 0.052). The frequency of the A allele in the AMS group was significantly lower than in the non-AMS group (25.3% vs 35.9%, respectively, OR = 0.607, 95%CI 0.377–0.975, P = 0.039, adjusted P = 0.078). In terms of genetic and clinical associations, a significant difference was detected in the SaO2 levels between the AG and GG genotypes of rs2044456 (P = 0.043) (Fig. 4A), while the SaO2 level in participants with the AG genotype of rs4953348 was significantly higher than participants with the GG genotype of rs4953348 (P = 0.005) (Fig. 4B).

### Medical verification

We further classified the AMS subjects (according to the rs4953348 allele) into A allele (AA plus AG, n = 24) and G allele (GG plus AG, n = 71) subgroups and compared pulmonary functions and the indicators for AMS diagnosis between the two subgroups. The FVC, MMF, and V25-50 in the G allele subgroup showed a trend of decrease in the A allele subgroup, but the HR and Vm-BA in the A allele subgroup indicated a trend of decrease compared that in the G allele subgroup. However, other physiological factors showed no significant differences between the two subgroups (Supplementary Table 5). After budesonide treatment, the results showed that the change values in FVC and HR were significantly higher in G allele subgroup, compared with the A allele subgroup (all of the adjust P values < 0.05) (Table 6).

## Discussion

High-altitude hypobaric hypoxia has a serious impact on a person’s physical condition and psychological state. Therefore, the body has developed a series of compensatory responses, with a sharp decline of the SaO2 level being the initial factor. Then subjects encounter the symptoms including headache induced by cerebral vasodilation, sleep disturbance due to decreased periodic respiration, along with anxiety^24^, which can further develop AMS. Subjects with AMS tend to have the hypoxic ventilator response with a lower arterial oxygen pressure, decreased vital capacity and a higher alveolar-arterial oxygen pressure difference (AaDO2)^23^,^25^,^26^,^27^. On the other hand, with the development of genomic technology, genetic polymorphisms associated with high altitudes have provided evidence for the important role of genes in the HIF oxygen-signaling pathway^22^,^28^. Mutations in HIF 2 (EPAS1) can induce AMS due to hypoxia.

In the present study, with the development of physiological changes, AMS incidence reached 54% among all subjects. A number of studies have analyzed AMS symptoms, however, the underlying mechanisms of AMS-related syndromes are still lacking. Thus, the present study extended previous findings by providing a systematic analysis of the changes in physical and psychological parameters and the underlying gene polymorphisms associated with AMS. In response to acute exposure to 3700 m, participants with AMS had poorer physical and mental health, higher HRs, MABPs and Tei indices and lower ETs. As indicated by the elevated Tei, HR and MABP increased, and ET decreased. A longitudinal regression analysis showed that these hemodynamic variables contributed to AMS development, consistent with the hypothesis of autonomic cardiovascular control leading to AMS^25^. With regard to spirometric parameter (FVC, MMF and V25-50 decreased), we found that the individuals with AMS had symptoms of AMS and pulmonary dysfunction (FVC, MMF and V25-50) decreased are consistent with appearance of early interstitial or alveolar edema evidenced by lesser Sao2 levels in AMS group in present study^25^ and changes in pulmonary function were associated with AMS after remaining at 3700 m for 1 day. Additionally, in individuals who are unable to afford and handle to increases in brain volume and cerebral blood flow induced by hypoxic vasodilatation through the displacement of cerebrospinal fluid the intracranial pressure could rise and further lead to AMS^9^,^29^,^30^,^31^. In this study, no significant differences in blood flow velocity of the MCA (MCAv) were detected between the AMS and non-AMS groups. MCAv likely plays a small role in AMS. Nevertheless, we found that increases in blood flow in the BA and VA may be responsible for AMS and were linked with headache due to cerebral vasodilation. Sympathetic excitability due to decline in SaO2 can cause augmented MABP, the excessive secretion of ACE, and further influence AMS development; thus, these factors can be used as indictors of AMS diagnosis. In particular, HR ≥ 82 beats/min, FVC ≤ 4.2 Lt, and Vm-BA ≥ 43 cm/s can be used as predictors for AMS diagnosis accompanied by high-altitude syndrome. Anxiety enhances sympathetic excitability and leads to physical changes, with elevated BP and HR being the most common ones. We used SAS to evaluate physical anxiety and found that anxiety correlated closely with AMS. Our data indicated that the individuals with AMS had more severe headache and insomnia than those without AMS, while the ones with headache were prone to insomnia and suffer from AMS. It suggested that the mental state had a strong effect on physical status, with anxiety being the most important factor for AMS-related headache and insomnia. The results from the genetic analysis of EPAS1 (rs2044456 and rs4953348) polymorphisms revealed different genotypes of AA, AG and GG in the recruited subjects. Although the genotypes and allele frequencies of rs2044456 showed no significant differences between the groups, rs4953348 polymorphisms were associated with AMS susceptibility, and the frequency of the A allele in the AMS group was significantly lower than in the non-AMS group. Thus, individuals with the AG genotype may be less vulnerable to AMS than those with the GG genotype, and the A allele can be considered as a protective factor against AMS. Nevertheless, the external environment and other factors (Age, race, and subjects with underlying diseases) should not be ignored. Notably, further analysis of the pulmonary function and other indicators for AMS diagnosis suggested that the G allele was related to the decreased spirometric parameter in a dominant locus, while the A allele associated with HR and Vm-BA in a dominant locus. Reduced spirometric parameter is the initial phenotype associated with the drop in SaO2 and the increase in AaDO2. Our previous study found that budesonide, an inhaled glucocorticoid, may generate similar but more potent local effects on the lung than dexamethasone, to improve the function of alveolar and airway epithelia, thus improving pulmonary function, increasing SpO2, and preventing acute mountain sickness^23^. In this study, the medication result showed that budesonide, may have more potent local effects on the lung in the AMS subjects with the G allele than the ones with the A allele, to improve the pulmonary function (alleviated the impairment of high altitude on forced vital capacity), consistent with our previous study^23^. It further verified that the G allele may contribute to restrictive ventilatory dysfunction in the subjects with AMS. Meanwhile, the A allele may serve as a protective factor against AMS through HR and Vm-BA compensation, which was further confirmed by the result that the SaO2 level in participants with the rs4953348 AA genotype was also higher than in the participants with the GG genotype.

## Conclusions

Our study presents three advances: first, we evaluated the physiological and psychological symptoms of AMS and the interrelationships between them after an acute ascent to high altitude. Second, the HR level, the LV Tei index, spirometric parameter, blood flow in the BA and VA, MABP, and ACE levels can be established as diagnostic indictors for AMS. In particular, HR, FVC, and Vm-BA can contribute to AMS prediction. Third, to explore the underlying molecular mechanism, we found that EPAS1 (rs2044456 and rs4953348) polymorphisms were associated with SaO2 level. The rs4953348 polymorphism had a physiological effect on AMS development, and the A allele can be considered as a protective factor against AMS through HR and Vm-BA compensation, while the G allele as a potential genetic marker to predict hypoxic pulmonary hypertension in AMS. However, in individuals contracting AMS at 2000–3000 m altitude the onset of symptoms occurs within 12 hr of arrival in 65% of subjects^32^. Due to large number of sample size and parameters we were able to collect all data by 24 hrs of high altitude induction. So further studies will be required to be conducted within 12 hr of high altitude exposure for getting better predictors for diagnosing AMS. Therefore, further studies with well-designed larger sample studies are needed in this field to further clarify the objective indicators and potential genotypes for AMS.

## Methods

### Participants and Study Design

A total of 176 non-Tibetan healthy young Chinese male lowland residents, aged between 19 and 27 years (mean years = 23.21 ± 3.65), were recruited for the study according to the inclusion and exclusion criteria. Before entering the high-altitude area, participants had no known clinical diseases, lived at 500 m and had not been exposed to high altitudes in the previous 12 months. Subjects with any one of the following conditions were excluded: migraine disease, autoimmune diseases, respiratory diseases, cardiovascular diseases, malignancy, liver and kidney dysfunction, active infection or a bad cold, and psychiatric disorders or neuroses that interfered with the completion of the questionnaires by a diplomate. The subjects did not take medication or receive any intervention before the ascent to high altitude.

All of the recruited participants were familiar with the purpose and procedures of the study and signed informed consent forms before the study. All study protocols were approved by the Ethics Committee of Xinqiao Hospital, the Second Clinic Medical College of the Third Military Medical University and was carried out in accordance with established national and institutional ethical guidelines regarding the involvement of human subjects and the use of human for research. Trial registration: Chinese Clinical Trial Registry, ChiCTR-RCS-12002232.

Demographic data were collected during recruitment. Baseline examinations were performed at 500 m (Chengdu). Then the 176 participants ascended to 3700 m (Lhasa) in 2 hours by airplane. The subjects didn’t have any strenuous exercise, drink, and smoke at the high altitude. Symptom questionnaires including physiological symptoms, a psychological scale and symptoms related to AMS, physiological parameters were recorded at 24 h after their arrival at 3700 m. Budesonide verification test was completed at 72 h.

Data were collected 24 h after their arrival at 3700 m. All participants completed the structured case report form (CRF) questionnaires, including self-reported demographic data, physiological symptoms, a psychological scale and symptoms related to AMS according to the LLS. Vital signs were also measured. Morning fasting venous blood was collected to test other physiological parameters and to isolate genomic DNA. Doppler ultrasound was implemented to measure the cardiac system and cerebral hemodynamic parameters. spirometric parameter was tested with a Sensor MedicsVmax229D pulmonary function instrument.

## Measurements

### Symptom scores for evaluating AMS, insomnia and anxiety

The LLS, which includes five self-report symptoms: headache, dizziness or light headedness, gastrointestinal symptoms (anorexia, nausea, or vomiting), All of the items were assessed on a 0 to 3 Likert scale, with 0 indicating none, 1 for light, 2 for moderate, and 3 for severe^3^,^4^,^5^,^33^,^34^. Clinical diagnosis of AMS was defined as a total score of the additive 5 items ≥3, including headache and one other symptom administered by physicians^3^,^4^,^5^,^17^,^18^,^33^,^34^. The Athens insomnia scale (AIS) is a self-assessment psychometric instrument that quantifies sleep difficulty. The severity of anxiety was diagnosed using the Self-rating Anxiety Scale (SAS).

### Vital signs

The second day after the participants arrived at 3700 m, blood pressure (BP), HR, and SaO2 levels were determined with a fingertip pulse oximeter (Nonin Onyx^®^ 9500, Nonin Medical, Inc., USA). All test items were measured in triplicate after the subjects had been seated for 30 min.

## Auxiliary examinations for high-altitude syndrome

### Cardiac function

Pulsed Doppler echocardiography (the frequency of the probe was 3.25 MHz) (VINGMED CFM-725, CHEMTECH Inc., USA) was used to investigate left or right ventricular systolic and diastolic functions. Values for left ventricular size, left and right ventricular ejection fractions (LVEF and RVEF, respectively), left and right ventricular fractional shortening (LVFs and RVFs, respectively), and the Tei index were recorded. In addition, right cardiac function and pulmonary artery pressure were detected.

### Pulmonary function

A pulmonary function instrument (Sensor MedicsVmax229D, USA) was used to measure spirometric parameter. Routine pulmonary function was measured when the subjects were seated. The detection index included forced expiratory volume in 1 second (FEV1), forced vital capacity (FVC), peak expiratory flow (PEF), vital capacity (25–75)% (V25, V50 and V75) and MMF. Each measurement was repeated twice, and the average value was used for statistical analysis.

### Brain function

TCD was used to measure the cerebral hemodynamic parameters of the middle cerebral artery (MCA), the basilar artery (BA), and the vertebral artery (VA). Doppler recordings were performed using a conventional ultrasound machine (Aloka SSD-α10, Japan) and 2.5 MHz Duplex probes. The peak systolic flow velocity (Vs), diastolic velocity (Vd), mean flow velocity (Vm), pulsatility index (PI), and resistance index (RI) were also recorded.

## Biological measurements

### Collection of blood samples and analysis of routine blood parameters and neuroendocrine

Morning fasting venous blood (4 ml) was collected with EDTA-K2, and 2 ml of the samples were used to assay routine blood parameters within 2 hours using a hematology analyzer and (Sysmex XE-2100, Japan). Additional blood samples (2 ml) were centrifuged at 4000 r/min for 10 min to separate serum and were stored at −80 °C until being used in assays. The concentrations of the neuroendocrine system, including epinephrine and norepinephrine, and the renin-angiotensin system (RAS), including angiotensin II (Ang II) and ACE, were measured with a fully automated biochemistry analyzer (Olympus Au2700, Japan) at Xinqiao Hospital.

### Genotyping

Genomic DNA was extracted from the fasting venous blood using the PUREGENE DNA Extraction kit (Gentra Systems, Inc., Minneapolis, MN, USA). The Primer premier 5 program (PREMIER Biosoft International, CA) was used to design the PCR primer sequences for genotyping EPAS1 polymorphisms. The EPAS1 (rs2044456) polymorphism was resolved using PCR sense (5′-GTTAGCTATGGTGCCCCAGAA-3′) and anti-sense (5′-GCAGCTCCTGGCTCAAAGCAT-3′) primers, which yielded a 200-bp product. Modified probes for rs2044456 were designed for combining with the probes (Sites A and G) to form 50-bp and 53-bp fragments. Meanwhile, the rs4953348 polymorphism was resolved using PCR sense (5′-CCAGGTCATAGAGTAAGTGTC-3′) and anti-sense (5′-TCTGGTCTTTGTTTGATGAGC-3′) primers, which yielded a 228-bp product. Modified probes for rs4953348 were designed for combining with the probes (Sites A and G) to form 57-bp and 60-bp fragments, respectively. The DNA sequencing reaction mixtures were conducted in a Thermo Arktik thermal cycler system using standard conditions. All genotyping was conducted using PCR and genomic DNA. Direct sequencing was performed for all samples with an automated sequencer (ABI Prisms^®^ 3130 Genetic Analyzer, Applied Biosystems, Life Technologies, USA).

### Medication

We took budesonide treatment to verify effects of the genotypes on the pulmonary function in the AMS subjects for 48 h. The detailed administration was performed according to Zheng CR etc study^23^.

### Statistical analysis

The results are presented as the means ± standard deviations (SD). Statistical analyses were performed using R software (http://www.R-project.org/). Changes in physiological parameters between 500 m and 3700 m were compared by paired-sample T-tests or Mann–Whitney U-test depend on the distribution. Independent T-tests were used to compare two values of normally distributed variables between the AMS group and the non-AMS group at 3700 m. The Mann-Whitney U-test was applied to evaluate differences between ordinal or non-normally distributed data. The Hardy-Weinberg equilibrium test and the χ2 test were used to assess the genotype distribution of loci and the differences in genotypes and allele frequencies in both the AMS group and the non-AMS group. An unpaired Student’s t-test was further used to compare the A allele (AA plus AG) and the G allele (GG plus AG) subgroups of the AMS group and the effects of budesonide treatment between the two subgroups. A forward, linear regression analysis was also performed to identify the physical factors with P < 0.05 between the AMS group and the non-AMS group, and spearman rank correlation analysis for cerebral hemodynamic parameters that are associated with LLS. Additionally, the variables related to LLS were considered potential risk factors in preliminary screening, and they were subjected to an adjusted multivariate logistic regression model to identify the adjusted independent predictors for AMS occurrence through backward stepwise regression. A logistic regression analysis was also performed to assess the relationships between the physical parameters and anxiety related to AMS symptoms and between the EPAS1 polymorphisms and AMS development. The physical parameters and symptoms were compared by paired-sample T test and the Mann–Whitney U-test, respectively, between 500 m and 3700 m. All multiple comparisons were conducted by Bonferroni correction.

## Additional Information

**How to cite this article**: Yu, J. *et al*. Analysis of High-altitude Syndrome and the Underlying Gene Polymorphisms Associated with Acute Mountain Sickness after a Rapid Ascent to High-altitude. *Sci. Rep.* **6**, 38323; doi: 10.1038/srep38323 (2016).

**Publisher's note:** Springer Nature remains neutral with regard to jurisdictional claims in published maps and institutional affiliations.

## Supplementary Material

Supplementary Dataset

## Figures and Tables

**Table 1:** Comparison of physiological parameters between 500 m and 3700 m.

Parameters	500 m	3700 m	P value (Bonferroni adjusted)
Physiological parameters (mean ± SD)			
SaO2 (%)	98.44 ± 0.92	88.94 ± 2.69	<0.01
HR (beats/min)	62.48 ± 8.98	78.74 ± 12.65	<0.01
Erythropoietic parameters (mean ± SD)			
[Hb] (g/L)	141.30 ± 10.68	145.91 ± 12.94	<0.05
HCT (L/L)	42.71 ± 3.22	45.32 ± 3.29	<0.05
MCHC (g/L)	331.05 ± 13.78	352.39 ± 8.13	<0.01
Left heart function (mean ± SD)			
EF (%)	62.96 ± 5.72	66.85 ± 3.04	<0.05
Neuroendocrine (mean ± SD)			
Epinephrine (ng/ml)	7.34 ± 4.30	10.21 ± 4.07	<0.01
Norepinephrine (ng/ml)	80.85 ± 42.29	126.36 ± 45.37	<0.01
Renin-angiotensin system (mean ± SD)			
Ang II (ng/ml)	0.24 ± 0.09	0.35 ± 0.20	<0.01
Ang-(1–7)(pg/ml)	22.95 ± 9.00	32.98 ± 14.81	<0.01

**Table 2:** Comparison of the demographic characteristics between the AMS and non-AMS groups.

Demographic factor	AMS group (n = 95)	Non-AMS group (n = 81)	P value
Age	22.78 ± 3.45	23.21 ± 3.96	0.181
Height (cm)	171.47 ± 4.77	171.23 ± 4.28	0.101
Weight (kg)	64.13 ± 7.78	62.17 ± 5.79	0.094
BMI (kg/m2)	21.92 ± 2.27	21.29 ± 1.73	0.069
Smoking			0.778
1	22 (23.2)	19 (23.4)	
2	50 (52.6)	44 (54.3)	
3	23 (24.2)	18 (22.3)	
Alcohol consumption			0.886
1	23 (24.2)	21 (25.9)	
2	3 (3.2)	5 (6.2)	
3	69 (72.6)	55 (67.9)	

Age and BMI are presented as the means ± standard deviations. High altitude exposure, smoking and alcohol consumption are expressed as numbers (percentages). BMI, body mass index; 1 = no smoking/drinking history, 2 = currently smoking/drinking and 3 = past smoking/drinking history.

**Table 3:** Logistic regression analysis for adjusted independent factors associated with AMS.

Adjusted independent factors	β-coefficient	Odds Ratio	95% CI	P value
Headache (yes)	0.98	3.15	1.63–5.30	<0.001
Anxiety (yes)	0.48	1.96	1.32–3.61	<0.001
Insomnia (yes)	0.78	2.43	1.45–4.09	<0.001
LV Tei index	0.61	1.33	1.16–3.42	0.041
ET (ms)	−0.09	0.91	0.86–0.96	0.007
HR (beats/min)	0.92	1.02	1.03–1.56	0.009
MABP (mm Hg)	0.83	1.82	1.65–3.71	0.015
ACE (ng/ml)	0.54	1.49	1.07–2.68	0.017
V_m-BA_ (cm/s)	0.95	2.84	1.94–3.57	0.038
V_m-VA_ (cm/s)	0.87	1.42	1.25–4.06	0.031
Spirometric Parameters				
FVC (Lt)	0.56	1.54	1.04–2.61	0.025
MMF (Lt)	0.74	2.67	1.76–3.19	0.012
V50 (Lt)	0.69	1.94	1.11–1.52	0.034
V25 (Lt)	0.36	2.08	1.59–2.83	0.028

**Table 4:** A logistic regression analysis of the predictors for AMS.

Factors	Odds Ratio (OR)	95% CI	*P* value
a: HR ≥ 82 beats/min	3.62	1.35–9.52	0.027
b: FVC ≤ 4.2 Lt	2.07	1.29–4.6	0.039
c: V_m-BA_ ≥ 43 cm/s	2.86	1.08–5.86	0.015
a + b + c	3.92	1.01–5.28	0.007

**Table 5:** Distribution of EPAS1 polymorphisms between the AMS group and the non-AMS group.

Gene loci	Genotype/Alleles	AMS group (n = 95)	Non-AMS group (n = 81)	*χ*^2^value	P value	OR (95% CI)^a^
rs2044456	GG	22 (23.2)	27 (33.3)			
	AG	59 (62.1)	42 (51.9)	1.706	0.191	1.602 (0.790–3.248)
	AA	14 (14.7)	12 (14.8)	0.344	0.557	1.351 (0.495–3.688)
	G	53 (55.8)	48 (59.3)			
	A	42 (44.2)	33 (40.7)	0.626	0.429	1.192 (0.771–1.843)
rs4953348	GG	51 (53.7)	32 (39.5)			
	AG	40 (42.1)	41 (50.6)	4.981	0.026	0.453 (0.226–0.908)
	AA	4 (4.2)	8 (9.9)	2.754	0.097	0.284 (0.064–1.256)
	G	71 (74.7)	52 (64.2)			
	A	24 (25.3)	29 (35.8)	4.256	0.039	0.607 (0.377–0.975)

95% CI: 95% confidence interval. A multivariate logistic regression model was used and adjusted for age, sex, BMI, smoking history, alcohol consumption, heart rate and oxygen saturation.

**Table 6:** Differences in the physiological parameters between A allele and G allele subgroups of the AMS subjects after budesonide treatment.

Parameter	A allele (n = 24)		G allele (n = 65)*	
	Post-treatment	Change values#	Post-treatment	Change values#
HR (beats/min)	79.53 ± 13.12	−3.53 ± 12.87†	82.26 ± 11.92	−5.54 ± 13.86
FVC (Lt)	4.28 ± 0.37	0.24 ± 0.43†	4.19 ± 0.41	0.42 ± 0.45
MMF (Lt)	4.39 ± 0.57	0.20 ± 0.64	4.33 ± 0.52	0.22 ± 0.39
V50 (Lt)	4.85 ± 0.58	0.29 ± 0.62	4.63 ± 0.62	0.38 ± 0.54
V25 (Lt)	2.15 ± 0.42	0.12 ± 0.45	2.09 ± 0.28	0.14 ± 0.32

^*^Six cases were excluded for poor quality of test.

^#^Change values between post-treatment and pretreatment of budesonide in the two subgroups.

^†^P < 0.05 for change values between A allele and G allele subgroups.

## References

[b1] Bolmont B., Thullier F. & Abraini J. H. Relationships between mood states and performances in reaction time, psychomotor ability, and mental efficiency during a 31-day gradual decompression in a hypobaric chamber from sea level to 8848 m equivalent altitude. Physiol Behav 71, 469–76 (2000). 11239664 10.1016/s0031-9384(00)00362-0

[b2] Montgomery A. B., Mills J. & Luce J. M. Incidence of acute mountain sickness at intermediate altitude. JAMA 261, 732–734 (1989). 2911169

[b3] Johnson T. S. & Rock P. B. Current concepts. Acute mountain sickness. N Eng J Med 319, 841–845 (1988). 10.1056/NEJM198809293191306 3045554

[b4] Maggiorini M., Buhler B., Walter M. & Oelz O. Prevalence of acute mountain sickness in the Swiss Alps. BMJ 301, 853–855 (1990). 2282425 10.1136/bmj.301.6756.853 PMC1663993

[b5] Roach R. C. & Hackett P. H. Frontiers of hypoxia research: acute mountain sickness. J Exp Biol 204, 3161–3170 (2001). 11581330 10.1242/jeb.204.18.3161

[b6] Burtscher M., Mairer K., Wille M. & Broessner G. Risk factors for high-altitude headache in mountaineers. Cephalalgia 31, 706–711 (2011). 21220379 10.1177/0333102410394678

[b7] Sahota P. K. & Dexter J. D. Transient recurrent situational insomnia associated with cluster headache. Sleep 16, 255–257 (1993). 8506459 10.1093/sleep/16.3.255

[b8] Kallenberg K. . Magnetic resonance imaging evidence of cytotoxic cerebral edema in acute mountain sickness. J Cereb Blood Flow Metab 27, 1064–1071 (2007). 17024110 10.1038/sj.jcbfm.9600404

[b9] Severinghaus J. W., Chiodi H., Brandstater B. & Hornbein T. F. Cerebral blood flow in man at high altitude. Role of cerebrospinal fluid pH in normalization of flow in chronic hypocapnia. Circ Res 19, 274–82 (1966). 5914844 10.1161/01.res.19.2.274

[b10] Agostoni P. . Acute high-altitude exposure reduces lung diffusion: data from the HIGHCARE Alps project. Respir Physiol Neurobiol 188, 223–228 (2013). 23619193 10.1016/j.resp.2013.04.005

[b11] Hohenhaus E., Paul A., McCullough R. E., Kücherer H. & Bärtsch P. Ventilatory and pulmonary vascular response to hypoxia and susceptibility to high altitude pulmonary oedema. Eur Respir J 8, 1825–1833 (1995). 8620946 10.1183/09031936.95.08111825

[b12] Selland M. A. . Pulmonary function and hypoxic ventilatory response in subjects susceptible to high-altitude pulmonary edema. Chest 103, 111–116 (1993). 8417862 10.1378/chest.103.1.111

[b13] Hultgren H. N., Grover R. F. & Hartley L. H. Abnormal circulatory responses to high altitude in subjects with a previous history of high-altitude pulmonary edema. Circulation 44, 759–770 (1971). 5115068 10.1161/01.cir.44.5.759

[b14] Hanaoka M. . Hypoxia-induced pulmonary blood redistribution in subjects with history of high-altitude pulmonary edema. Circulation 101, 1418–1422 (2000). 10736286 10.1161/01.cir.101.12.1418

[b15] Dong J. Q. . Anxiety correlates with somatic symptoms and sleep status at high altitudes. Physiolo Behav 15, 112–113:23–31 (2013). 10.1016/j.physbeh.2013.02.001 23403037

[b16] Hornbein T. F., Townes B. D., Schoene R. B., Sutton J. R. & Houston C. S. The cost to the central nervous system of climbing to extremely high altitude. N Engl J Med 321, 1714–1719 (1989). 2512483 10.1056/NEJM198912213212505

[b17] Bahrke M. S. & Hale B. Effects of altitude on mood, behavior and cognitive functioning. Sports Med 16, 97–125 (1993). 8378672 10.2165/00007256-199316020-00003

[b18] Watson D. . Testing a tripartite model: II. Exploring the symptom structure of anxiety and depression in student, adult, and patient samples. J Abnorm Psychol 104, 15–25 (1995). 7897037 10.1037//0021-843x.104.1.15

[b19] Imray C., Wright A., Subudhi A. & Roach R. Acute mountain sickness: pathophysiology, prevention, and treatment. Prog Cardiovasc Dis 52, 467–484 (2010). 20417340 10.1016/j.pcad.2010.02.003

[b20] Sahota P. K. & Dexter J. D. Transient recurrent situational insomnia associated with cluster headache. Sleep 16, 255–257 (1993). 8506459 10.1093/sleep/16.3.255

[b21] A Bigham M. . Identifying signatures of natural selection in Tibetan and Andean populations using dense genome scan data. PLoS Genet 6, e1001116 (2010). 20838600 10.1371/journal.pgen.1001116 PMC2936536

[b22] Simonson T. S. . Genetic evidence for high-altitude adaption in Tibet. Science 329, 72–75 (2010). 20466884 10.1126/science.1189406

[b23] Zheng C. R. . Inhaled budesonide and oral dexamethasone prevent acute mountain sickness. Am J Med 127, 1001–1009.e2 (2014). 24784698 10.1016/j.amjmed.2014.04.012

[b24] Roth W. T. . High altitudes, anxiety, and panic attacks: is there a relationship? Depress Anxiety 16, 51–58 (2002). 12219335 10.1002/da.10059

[b25] Anholm J. D., Houston C. S. & Hyers T. M. The relationship between acute mountain sickness and pulmonary ventilation at 2,835 meters (9,300 ft). Chest 75, 33–36 (1979). 421519 10.1378/chest.75.1.33

[b26] Agostoni P. . Acute high-altitude exposure reduces lung diffusion: data from the HIGHCARE Alps project. Respir Physiol Neurobiol 188, 223–8 (2013). 23619193 10.1016/j.resp.2013.04.005

[b27] Bärtsch P. . Prevention of high-altitude pulmonary edema by nifedipine. N Engl J Med 18, 1284–9 (1991). 10.1056/NEJM199110313251805 1922223

[b28] Bigham A. W. . Identifying positive selection candidate loci for high-altitude adaptation in Andean populations. Hum Genomics 4, 79–90 (2009). 20038496 10.1186/1479-7364-4-2-79 PMC2857381

[b29] Lanfranchi P. A. Autonomic cardiovascular regulation in subjects with acute mountain sickness. Am J Physiol Heart Circ Physiol 289, H2364–72 (2005). 16055524 10.1152/ajpheart.00004.2005

[b30] Hackett P. H. & Roach R. C. High-altitude illness. N Engl J Med 345, 107–114 (2001). 11450659 10.1056/NEJM200107123450206

[b31] Morocz I. A. . Volumetric quantification of brain swelling after hypobaric hypoxia exposure. Exp Neurol 168, 96–104 (2001). 11170724 10.1006/exnr.2000.7596

[b32] Honigman B. . Acute mountain sickness in a general tourist population at moderate altitude. Ann Intern Med 118, 587–592 (1993). 8452324 10.7326/0003-4819-118-8-199304150-00003

[b33] Riepl R. L. . Influence of acute exposure to high altitude on basal and postprandial plasma levels of gastroenteropancreatic peptides. PLoS One 7, e44445 (2012). 22970220 10.1371/journal.pone.0044445 PMC3435278

[b34] Wu T. Y. . Smoking, acute mountain sickness and altitude acclimatisation: a cohort study. Thorax 67, 914–9 (2012). 22693177 10.1136/thoraxjnl-2011-200623

