# Supplementary material for: Analysis of High-altitude Syndrome and the Underlying Gene Polymorphisms Associated with Acute Mountain Sickness after a Rapid Ascent to High-altitude
Source: Sci Rep. 2016 Dec 16;6:38323. doi: 10.1038/srep38323 (PMC5159877; doi:10.1038/srep38323)

**Analysis of High-altitude Syndrome and the Underlying Gene Polymorphisms Associated with Acute Mountain Sickness after a Rapid Ascent to High-altitude**

Jie Yu, Ying Zeng, Guozhu Chen, Shizhu Bian, Youzhu Qiu, Xi Liu, Baida Xu, Pan Song, Jihang Zhang, Jun Qin*^2^, Lan Huang*^1^

Institute of Cardiovascular Diseases of PLA, Xinqiao Hospital, Third Military Medical University, Chongqing, China.

*1 Correspondence to: Institute of Cardiovascular Diseases of PLA, Xinqiao Hospital, Third Military Medical University, Chongqing, China, 400037. Telephone / Fax: +86-23-68755601. E-mail: gyhl260@outlook.com

*^2^ Correspondence to: Institute of Cardiovascular Diseases of PLA, Xinqiao Hospital, Third Military Medical University, Chongqing, China, 400037. Telephone / Fax: +86-23-68755601. E-mail: dsjyyxj163@outlook.com

**Clinical Trial Registry:** Chictr.org; URL: www.chictr.org; No.: ChiCTR-RCS-12002232.

| **Supplementary Table 1. Comparison of physiological parameters between 500 m and 3700 m.** | | | |
| --- | --- | --- | --- |
| Parameters | 500 m | 3700 m | Pvalue (Original) |
| **Physiological parameters** |  |  |  |
| SaO_2_ (%) | 98.44±0.92 | 88.94±2.69 | < 0.001 |
| HR (beats/min) | 62.48±8.98 | 78.74±12.65 | < 0.001 |
| **Erythropoietic parameters** |  |  |  |
| [Hb] (g/L) | 141.30±10.68 | 145.91±12.94 | < 0.001 |
| RBC (10^12^) | 4.66±0.59 | 4.98±0.39 | < 0.01 |
| HCT (L/L) | 42.71±3.22 | 45.32±3.29 | < 0.001 |
| MCV (fl) | 91.35±7.81 | 90.44±6.44 | >0.05 |
| MCH (pg) | 30.37±3.05 | 31.88±2.30 | >0.05 |
| MCHC (g/L) | 331.05±13.78 | 352.39±8.13 | < 0.001 |
| **Left heart function** |  |  |  |
| LA (mm) | 30.85±1.62 | 29.87±1.66 | < 0.05 |
| LV (mm) | 46.40±2.52 | 46.36±1.94 | >0.05 |
| EF (%) | 62.96±5.72 | 66.85±3.04 | < 0.001 |
| FS (%) | 35.19±3.93 | 36.08±3.35 | >0.05 |
| SV (ml) | 64.48±10.64 | 67.73±8.33 | < 0.05 |
| SVI (ml/m^2^) | 37.92±5.91 | 39.90±5.12 | < 0.05 |
| CO (L/min) | 4.06±0.84 | 5.34±1.04 | < 0.01 |
| CI (L/m^2^) | 2.39±0.50 | 3.15±0.65 | < 0.01 |
| Mitral E/A ratio | 1.99±0.61 | 1.67±0.57 | < 0.01 |
| ET (ms) | 295.9±29.47 | 272.15±25.65 | < 0.01 |
| Tei index | 0.40±0.11 | 0.48±0.12 | < 0.01 |
| **Right heart function** |  |  |  |
| RA (mm) | 34.11±2.00 | 36.00±2.00 | < 0.01 |
| RV (mm) | 35.77±1.91 | 33.45±2.25 | < 0.01 |
| Tricuspid E/A ratio | 1.64±0.31 | 1.28±0.21 | < 0.05 |
| Tricuspid E/A ratio | 1.64±0.31 | 1.28±0.21 | < 0.05 |
| Tei index | 0.19±0.05 | 0.27±0.66 | < 0.05 |
| mPAP (mm Hg) | 14.29±3.46 | 23.08±6.97 | < 0.01 |
| **Pulmonary ventilation** |  |  |  |
| FVC (Lt) | 4.36±0.47 | 4.03±0.49 | < 0.05 |
| FEV1 (Lt) | 3.68±0.34 | 3.62±0.37 | >0.05 |
| PEF (Lt) | 9.24±1.07 | 9.30±1.26 | >0.05 |
| MMF (Lt) | 4.88±0.77 | 4.39±0.59 | < 0.05 |
| V75 (Lt) | 8.09±1.42 | 8.02±1.45 | >0.05 |
| V50 (Lt ) | 5.34±0.74 | 4.87±0.69 | < 0.05 |
| V25 (Lt) | 2.32±0.26 | 2.13±0.27 | < 0.05 |
| **Neuroendocrine** |  |  |  |
| Epinephrine (ng/ml) | 7.34±4.30 | 10.21±4.07 | < 0.001 |
| Norepinephrine (ng/ml) | 80.85±42.29 | 126.36±45.37 | < 0.001 |
| EPO | 3.31±1.20 | 3.8510±1.42 | < 0.05 |
| **Renin-angiotensin system** |  |  |  |
| Renin(pg/ml) | 33.42±13.96 | 32.94±17.42 | >0.05 |
| AngⅡ(ng/ml) | 0.24±0.09 | 0.35±0.20 | < 0.001 |
| Ang-(1-7)(pg/ml) | 22.95±9.00 | 32.98±14.81 | < 0.001 |
| ACE (ng/ml) | 0.61±0.22 | 0.70±0.30 | < 0.05 |
| ACE2(pg/ml) | 778.46±160.94 | 875.89±192.14 | < 0.005 |
| **Symptoms** |  |  |  |
| Headache (yes) | 2 (1.1) | 114 (64.8) | < 0.01 |
| Insomnia (yes) | 4 (2.3) | 86 (48.9) | < 0.01 |
| Anxiety (yes) | 1(0.6) | 53 (30.1) | < 0.01 |

| **Supplementary Table 2. Comparison of cerebral hemodynamics between 500 and 3700 m.** | | | | |
| --- | --- | --- | --- | --- |
| Parameter | 500 m | 3700 m | P value (original) | P value (adjust) |
| MCA |  |  |  |  |
| Vs (cm / s) | 92±10 | 96±9 | < 0.01 | >0.05 |
| Vd (cm / s) | 40±8 | 43±8 | < 0.01 | >0.05 |
| Vm (cm / s) | 59±9 | 62±11 | < 0.01 | >0.05 |
| PI | 0.86±0.15 | 0.82±0.11 | < 0.01 | >0.05 |
| RI | 0.55±0.12 | 0.53±0.10 | < 0.01 | >0.05 |
| BA |  |  |  |  |
| Vs (cm / s) | 65±14 | 67±13 | < 0.05 | >0.05 |
| Vd (cm / s) | 25±8 | 29±7 | < 0.01 | >0.05 |
| Vm (cm / s) | 39±9 | 42±8 | < 0.01 | >0.05 |
| PI | 1.12±0.21 | 0.91±0.24 | < 0.01 | >0.05 |
| RI | 0.63±0.10 | 0.56±0.11 | < 0.01 | >0.05 |
| VA |  |  |  |  |
| Vs (cm / s) | 48±9 | 50±8 | < 0.05 | >0.05 |
| Vd (cm / s) | 22±3 | 25±4 | < 0.01 | >0.05 |
| Vm (cm / s) | 33±6 | 37±5 | < 0.01 | >0.05 |
| PI | 0.85±0.16 | 0.72±0.13 | < 0.01 | >0.05 |
| RI | 0.56±0.07 | 0.50±0.06 | < 0.01 | >0.05 |
|  |  |  |  |  |

| **Supplementary Table 3. Differences in clinical characteristics between the AMS and non-AMS groups at 3700 m.** | | | | |
| --- | --- | --- | --- | --- |
| Item | AMS group (n=95) | Non-AMS group  (n=81) | P value  (Original) | P value  (adjust) |
| **Symptoms(%)** | | | |  |
| Headache (yes) | 95 (100) | 16 (19.7) | < 0.001 | < 0.01 |
| Dizziness (yes) | 89 (93.7) | 19 (23.5) | < 0.001 | < 0.01 |
| Gastrointestinal symptoms (yes) | 32 (33.6) | 5 (6.2) | < 0.001 | < 0.01 |
| Difficulty sleeping (yes) | 70 (73.7) | 20 (24.7) | < 0.001 | < 0.01 |
| Fatigue (yes) | 85 (89.5) | 25 (30.9) | < 0.001 | < 0.01 |
| Insomnia (yes) | 63 (66.3) | 19 (23.5) | < 0.001 | < 0.01 |
| Anxiety (yes) | 39 (41.1) | 13 (16.0) | < 0.001 | < 0.01 |
| LLS | 4.57±1.61 | 1.42±0.94 | < 0.001 | < 0.01 |
| **Physiological parameters (mean ± SD)** | | | |  |
| SaO_2_ (%) | 88.54±3.27 | 89.41±2.74 | < 0.01 | - |
| HR (beats/min) | 82.03±12.77 | 75.45±11.63 | < 0.01 | - |
| SBP (mmHg) | 122.69±13.41 | 118.77±11.00 | >0.05 | - |
| DBP (mmHg) | 80.37±8.97 | 77.76±10.08 | >0.05 | - |
| MABP (mmHg) | 94.48±9.40 | 91.43±9.56 | < 0.05 | - |
| **Left heart function (mean ± SD)** | | | |  |
| LV (mm) | 46.61±2.68 | 47.09±1.97 | >0.05 | - |
| LA (mm) | 29.10±2.34 | 29.79±1.67 | >0.05 | - |
| EF (%) | 66.96±4.36 | 66.83±3.99 | >0.05 | - |
| FS (%) | 37.54±3.67 | 36.71±3.01 | >0.05 | - |
| SV (ml) | 67.45±8.72 | 68.84±7.96 | >0.05 | - |
| SVI | 39.43±5.61 | 40.80±4.55 | >0.05 | - |
| CO | 5.53±1.11 | 5.19±1.00 | >0.05 | - |
| CI | 3.23±0.68 | 3.09±0.62 | >0.05 | - |
| Mitral E/A ratio | 1.68±0.70 | 1.66±0.44 | >0.05 | - |
| ET (ms) | 255.75±21.39 | 288.54±27.90 | < 0.01 | - |
| Tei index | 0.54±0.12 | 0.42±0.09 | < 0.01 | - |
| **Right heart function (mean ± SD)** | | | |  |
| RA (mm) | 36.41±2.20 | 35.87±1.96 | >0.05 | - |
| RV (mm) | 33.41±2.35 | 33.92±2.22 | >0.05 | - |
| Tricuspid E/A ratio | 1.24±0.26 | 1.31±0.18 | >0.05 | - |
| Tei index | 0.31±0.07 | 0.24±0.05 | < 0.01 | - |
| mPAP (mmHg) 24.21±4.86 23.87±4.34 >0.05  Brain function (mean ± SD) | | | |  |
| PI _MCA_ | 0.79±0.13 | 0.80±0.14 | >0.05 | - |
| RI _MCA_ | 0.54±0.03 | 0.53±0.04 | >0.05 | - |
| Vs_BA (cm / s) | 68±16 | 65±13 | < 0.05 | - |
| Vd_BA (cm / s) | 32±7 | 30±8 | < 0.05 | - |
| Vm_BA (cm / s) | 43±9 | 41±10 | >0.05 | - |
| Vd_VA (cm / s) | 25±5 | 24±5 | < 0.05 | - |
| Vm_VA (cm / s) | 36±7 | 35±6 | >0.05 | - |
| PI _VA_ | 0.72±0.11 | 0.75±0.13 | < 0.01 | - |
| RI _VA_ | 0.48±0.06 | 0.50±0.08 | < 0.01 | - |
| ΔVs_VA (cm / s) | 0.00(11.00) | -1.00 (9.00) | < 0.05 | - |
| ΔVd_VA (cm / s) | -1.00 (7.00) | -2.00 (8.00) | < 0.01 | - |
| ΔVm_VA (cm / s) | -1.00 (8.00) | -2.00 (9.00) | < 0.05 | - |
| AI_VA_ % | -2.4(17.5) | -5.6 (20.5) | < 0.05 | - |
| **Neuroendocrine (mean ± SD)** | | | |  |
| Epinephrine (ng/ml) | 11.09±4.14 | 9.06±3.75 | < 0.05 | - |
| Norepinephrine(ng/ml) | 137.42±41.75 | 112.09±46.50 | < 0.05 | - |
| EPO | 4.81±1.43 | 4.27±1.416 | >0.05 | - |
| **RAS (mean ± SD)** | | | |  |
| Renin(pg/ml) | 33.41±18.31 | 31.95±16.88 | >0.05 | - |
| AngⅡ(ng/ml) | 0.38±0.06 | 0.30±0.06 | >0.05 | - |
| Ang-(1-7)(pg/ml) | 34.34±14.55 | 31.22±15.21 | >0.05 | - |
| ACE (ng/ml) | 0.79±0.27 | 0.58±0.29 | < 0.05 | - |
| ACE2(pg/ml) | 881.21±207.12 | 869.03±174.04 | >0.05 | - |
| Physiological parameters were compared by the paired-sample T test between the AMS and non-AMS groups. The physical and psychological symptoms were compared by the Mann–Whitney U-test. Incidences of symptoms are expressed as numbers (percentages). The sign "-" indicate no statistical difference. | | | | |

| **Supplementary Table 4. Relationship between LLS and cerebral hemodynamic parameters at 3700 m.** | | | |
| --- | --- | --- | --- |
|  |  | Spearman r | P value |
| MCA | PI _MCA_ | -0.062 | 0.029 |
|  | RI_MCA_ | -0.127 | 0.027 |
| BA | Vm_BA | -0.012 | 0.013 |
|  | Vs_BA | 0.041 | 0.018 |
|  | Vd_BA | 0.022 | 0.004 |
|  | PI _BA_ | -0.136 | 0.005 |
|  | RI _BA_ | -0.148 | 0.002 |
| VA | Vm_VA | 0.153 | 0.014 |
|  | Vd_VA | 0.079 | 0.001 |
|  | PI _VA_ | -0.057 | 0.001 |
|  | RI _VA_ | -0.07 | <0.001 |
|  | ΔVm_VA | 0.068 | <0.001 |
|  | AI_VA_ | 0.01 | <0.001 |
|  | ΔVd_VA | 0.028 | <0.001 |
|  | ΔVs_VA | 0.002 | 0.001 |

| **Supplementary Table 5. Physiological parameters of the AMS group based on the alleles of the rs4953348 polymorphism** | | | | |  |
| --- | --- | --- | --- | --- | --- |
| Parameter | A allele | G allele | | P value | |
|  | (AA plus AG, n = 24) | (GG plus AG, n = 71) | |  |  |
| FVC (Lt) | 4.05±0.58 | 3.78±0.34 | 0.007 | |  |
| MMF (Lt) | 4.28±0.63 | 4.12±0.43 | 0.034 | |  |
| V50 (Lt) | 4.57±0.69 | 4.25±0.57 | 0.027 | |  |
| V25 (Lt ) | 2.04±0.37 | 1.96±0.22 | 0.036 | |  |
|  |  |  |  | |  |
| RV (mm Hg) | 33.21±3.57 | 33.67±2.15 | 0.45 | |  |
| LV Tei index | 0.53±0.09 | 0.55±0.15 | 0.529 | |  |
| ET (ms) | 255.65±21.71 | 256.04±20.35 | 0.937 | |  |
| HR (beats / min) | 83.07±12.05 | 87.79±13.61 | 0.036 | |  |
| MABP (mm Hg) | 93.99±10.7 | 95.91±8.9 | 0.388 | |  |
| ACE (ng / ml) | 0.78±0.30 | 0.81±0.23 | 0.607 | |  |
| V_m-BA_ (cm / s) | 42±10 | 46±6 | 0.021 | |  |
| V_m-VA_ (cm / s) | 36±6 | 39±8 | 0.096 | |  |
|  |  |  |  | |  |

**Supplementary Figure 1.** Record of the pulsed Doppler echocardiography showing cardiac function at 500 m and 3700 m. A: The spectrum of pulmonary arterial flow; B: the spectrum of tricuspid flow; C: the spectrum of right atrial diameter.


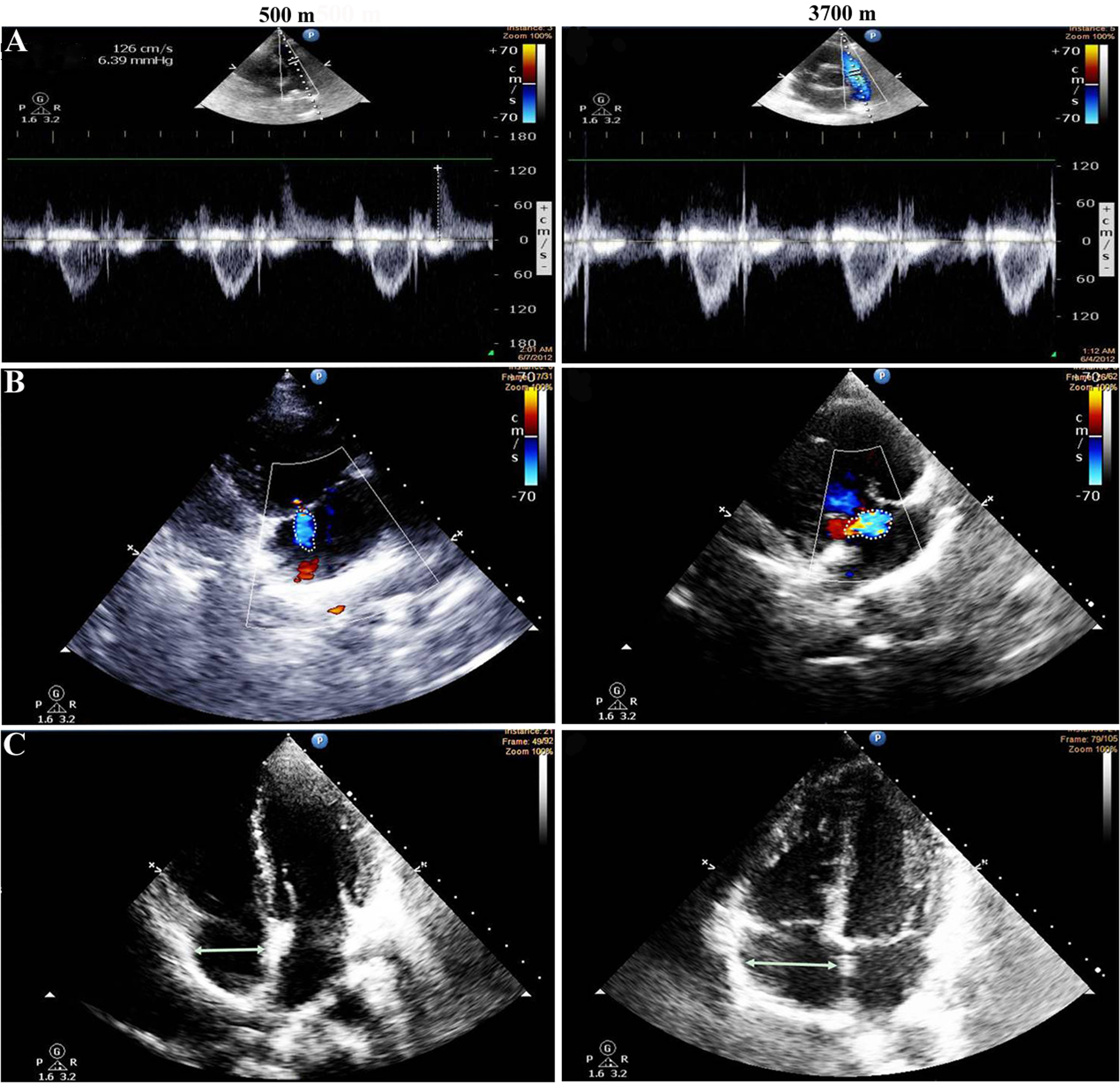


**Supplementary Figure 2.** Illustration of a pulsed Doppler diagram showing the measurement of each parameter for calculating the Tei index. A: a = isovolumetric contraction time (ICT) + ejection time (ET) + isovolumetric relaxation time (IRT); b= ET. Tei index = (ICT+IRT) / ET = (a-b)/b. B: Pulsed Doppler graph revealing the prolonged IRT and shortened ET, yielding an increased Tei index, in the AMS group at 3700 m. C: Pulsed Doppler diagram displaying the maintained IRT and ET, yielding noTei index alteration, in the non-AMS group(at 3700 m).


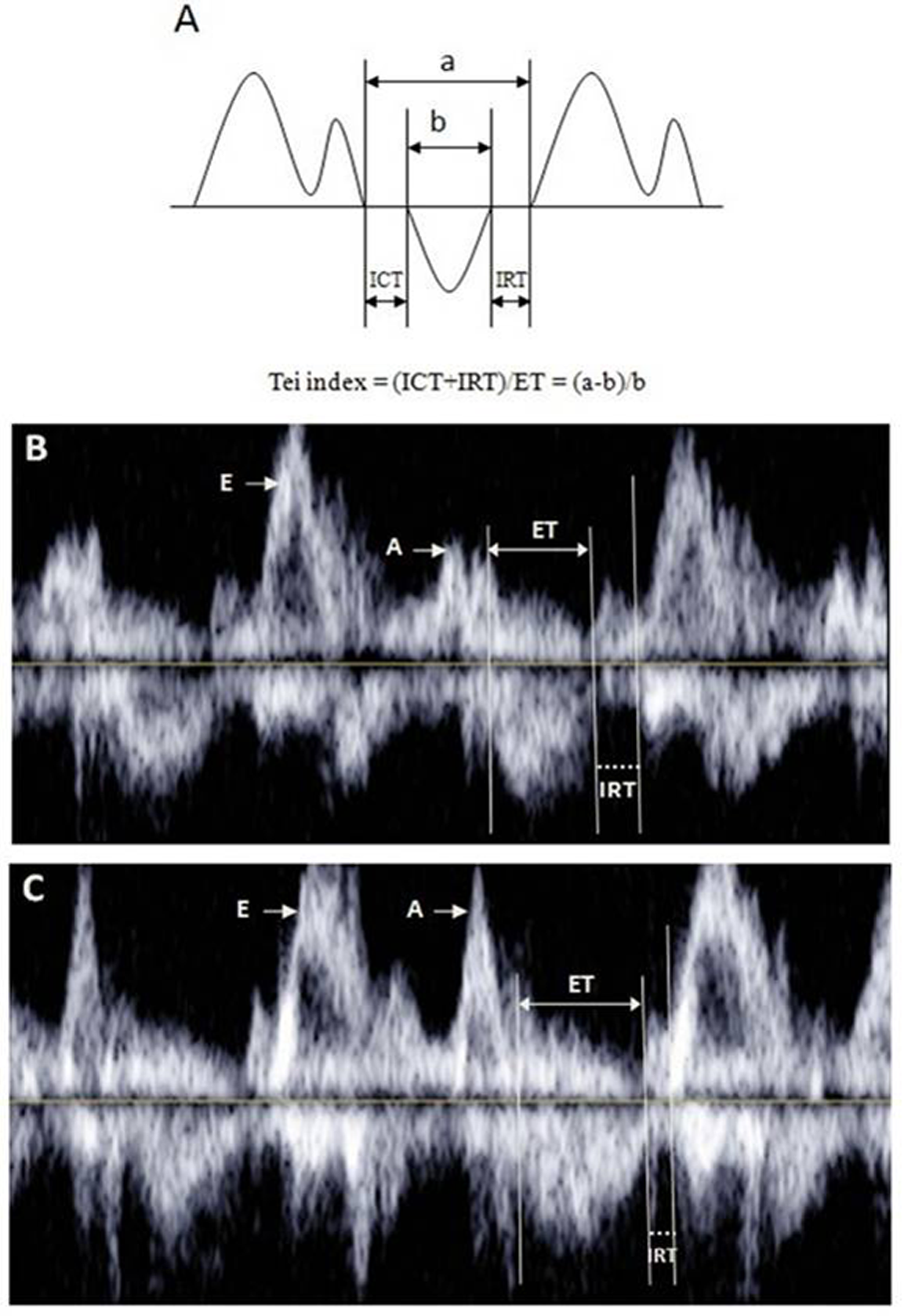


**Supplementary Figure 3.** A: Charts a1, a2, and a3 represent the genetic sequencing of genotypes AA, AG, and GG of rs2044456, respectively. B: Charts a1, a2, and a3 represent the genetic sequencing of genotypes AA, AG, and GG of rs4953348, respectively.


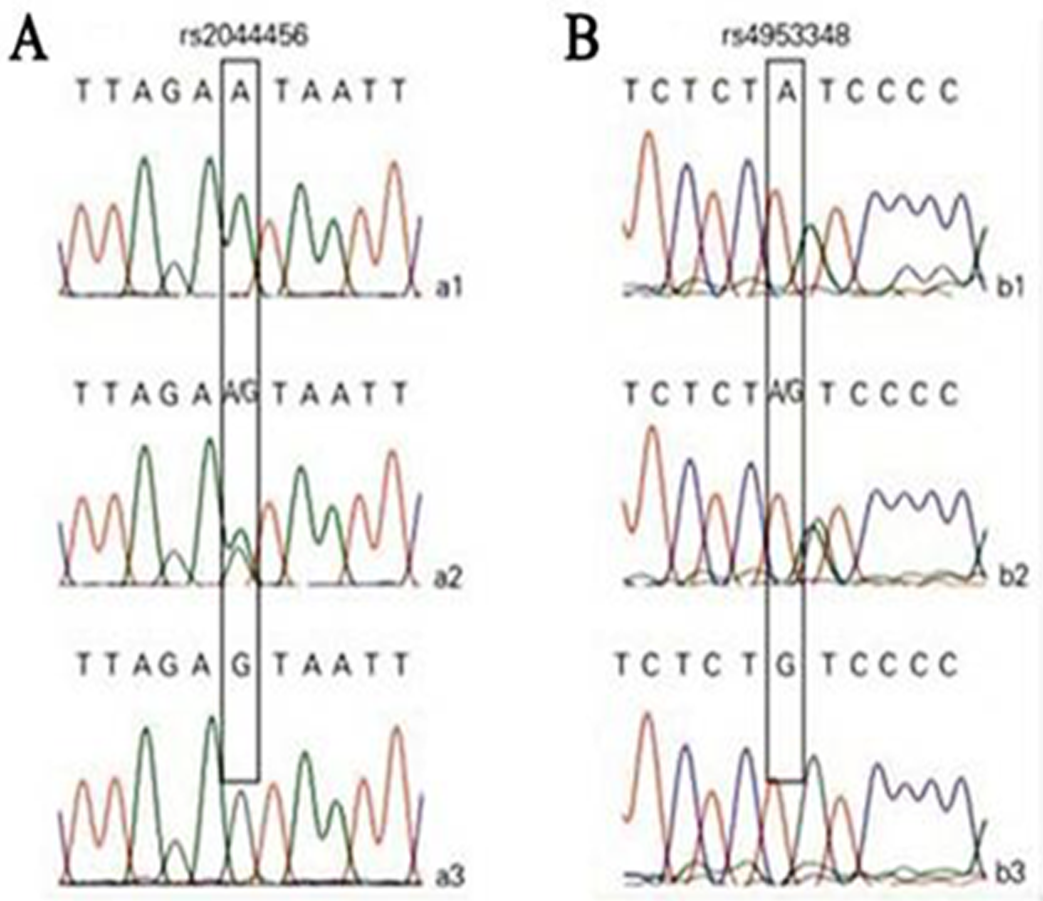

Supplement: Supplementary Dataset [file srep38323-s1.doc]
